# Fluid‐based augmentation of magnetic resonance visualization of interventional devices

**DOI:** 10.1002/acm2.13407

**Published:** 2021-08-28

**Authors:** Jens Kübler, Petros Martirosian, Johann Jacoby, Georg Gohla, Moritz T. Winkelmann, Konstantin Nikolaou, Rüdiger Hoffmann

**Affiliations:** ^1^ Department of Diagnostic and Interventional Radiology University Hospital of Tübingen Tübingen Germany; ^2^ Section on Experimental Radiology University Hospital of Tübingen Tübingen Germany; ^3^ Institute of Clinical Epidemiology and Applied Biometry University Hospital of Tübingen Tübingen Germany

**Keywords:** contrast media, interventional devices, MR‐guided intervention, percutaneous interventions

## Abstract

**Purpose:**

To evaluate the transient artifact augmentation of microtubes in magnetic resonance imaging by fluid injection.

**Methods:**

Twenty‐one fluorinated ethylene propylene catheters (inner diameter 760 μm) were filled with three different contrast media at various concentrations (Ferucarbotran, Resovist®, Bayer Schering Pharma; Manganese dichloride, MnCl2, Sigma‐Aldrich; Gadobutrol, Gadovist®, Bayer Schering Pharma). Artifact appearance was determined in an ex vivo phantom at 1.5 T using three different sequences: T1‐weighted three‐dimensional volume interpolated breath‐hold examination, T2‐weighted turbo spin echo, and T1‐weighted fast low angle shot. Catheter angulation to the main magnetic field (B0) was varied. Influence of parameters on artifact diameters was assessed with a multiple linear regression similar to an analysis of variance.

**Results:**

Artifact diameter was significantly influenced by the contrast agent (*p* < 0.001), concentration of the contrast agent (*p* < 0.001), angulation of the phantom to B0 with the largest artifact at 90° (*p* < 0.001), and encoding direction with a larger diameter in phase encoding direction (PED, *p* < 0.001). Mean artifact diameters at 90° angulation to B0 in PED were 18.5 ± 5.4 mm in 0.5 mmol/ml Ferucarbotran, 8.7 ± 2.5 mm in 1 mmol/ml Gadobutrol, and 11.6 ± 4.6 mm in 5 mmol/ml MnCl_2_.

**Conclusions:**

Fluid‐based contrast agents might be applied to interventional devices and thus temporarily augment the artifact ensuring both visibility and safe navigation.

## 1 INTRODUCTION

Magnetic resonance imaging (MRI) guidance is an alternative modality for percutaneous interventions, such as biopsies or ablation procedures.^1^, ^2^, ^3^ Technical advantages over the more commonly used modalities computed tomography and ultrasound comprise near real‐time fluoroscopic imaging, higher sensitivity in depicting small parenchymal lesions, free selection of imaging planes, absence of ionizing radiation, and monitoring of thermal effects during ablation procedures.^4^, ^5^, ^6^ Several requirements concerning the device visibility under MRI‐guidance have to be fulfilled to enable a safe device placement. However, these requirements can change during the procedure. While a distinct MRI artifact of the device allows a clear visualization during positioning under MRI fluoroscopy, a large artifact can obscure relevant anatomical structures after positioning and, for example, impede therapy assessment during tumor ablation.^7^ Several publications have addressed this topic with different technical approaches.^8^, ^9^, ^10^ Eibofner et al. suggested an artifact augmentation of an MRI‐compatible device with the artifact size being adjustable by changing the sequence parameters.^11^ However, this approach requires a modification of the applicator with usage of additional materials. Several devices for MR‐guided procedures are equipped with submillimeter channels. For percutaneous thermoablation, most applicators contain a fluid channel for shaft cooling during ablation. Theoretically, these channels can temporarily be filled with fluids to enhance the visualization of the device.

The aim of our phantom study was, therefore, to provide a comprehensive assessment of the transient artifact augmentation caused by a fluid‐filled microtube.

## 2 MATERIALS AND METHODS

### 2.1 Materials

Fluorinated ethylene propylene (FEP) catheters (Vasofix, B. Braun Melsungen AG, Germany) with an inner diameter of 760 μm (length: 33 mm, outer diameter: 1100 μm) were used for artifact measurements. Catheters were filled with fluid contrast media solution using a 2 ml syringe. Attention was paid to avoid accidental injection of air bubbles. The catheter was then sealed to prevent diffusion of the injected fluid into the surrounding water.

The following contrast agents were used: (1) Gadobutrol (Gadovist®, Bayer Schering Pharma, Berlin, Germany) at a stock solution of 1 mmol/ml; (2) Ferucarbotran (Resovist®, Bayer Schering Pharma) at a stock solution of 0.5 mmol/ml; and (3) Manganese dichloride (MnCl_2_, Sigma‐Aldrich, St. Louis, MO, USA) at a stock solution of 5 mmol/ml. A serial dilution of all contrast agents in water was generated (100%, 80%, 60%, 40%, 20%, 10%, and 5% of the stock solution). Dilutions are summarized in Table 1. For each contrast agent, the following experimental setup was applied: Seven catheters filled with contrast medium in the described descending concentration and one reference catheter filled with water were parallel mounted in a clamping device. This device was positioned in an ex vivo test phantom consisting of a Plexiglas box filled with water.

**TABLE 1:** Serial dilutions of contrast media

	100%	80%	60%	40%	20%	10%	5%
Gadobutrol (mmol/ml)	1	0.8	0.6	0.4	0.2	0.1	0.05
Ferucarbotran (mmol/ml)	0.5	0.4	0.3	0.2	0.1	0.05	0.025
MnCl_2_ (mmol/ml)	5	4	3	2	1	0.5	0.25

To illustrate possible application in clinical practice, the cooling circulation system of an MRI‐compatible therapeutic microwave antenna (Disposable Microwave Therapeutic Antenna, 14G, 15 cm, Nanjing ECO Microwave System Co., Ltd., Nanjing, China) was filled with fluid contrast media in a similar manner.

### 2.2 Measurements

Measurements were performed with a 1.5 T wide‐bore MRI scanner (MAGNETOM Aera, Siemens Healthcare, Erlangen, Germany) and a four‐channel body array surface coil. Artifact measurements were performed using three different pulse sequences: (1) 3D T1‐weighted *volume interpolated breath‐hold examination (VIBE)* sequence with chemically selective fat‐saturation pulse (TR = 4.93 ms, TE = 1.94 ms, FA = 10°, BW = 260 Hz/pixel, ST = 1 mm, FOV = 320×320 mm, matrix = 320 × 320). (2) 2D T2‐weighted *turbo spin echo (TSE)* sequence (TR = 5420 ms, TE = 65 ms, FA = 150°, ETL = 10, BW = 195 Hz/pixel, ST = 4 mm, FOV = 320×320 mm, matrix = 320 × 320). (3) 2D T1‐weighted *fast low angle shot (FLASH)* sequence with periodic chemically selective fat‐saturation pulses (TR = 255 ms, TE = 2.46 ms, FA = 70°, BW = 300 Hz/pixel, ST = 4 mm, FOV = 320 × 320 mm, matrix = 320 × 320).

**FIGURE 1:**
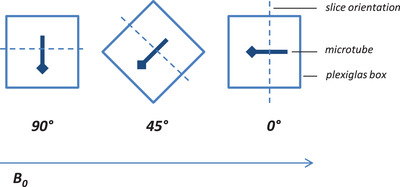
Experimental setup. Microtubes were filled with contrast media and mounted horizontally in a phantom consisting of a plexiglas box filled with water. The angulation to the magnetic field (B0) was varied between 0°, 45°, and 90°. Slice orientation was adjusted (dashed line).

Overall, three different contrast media (Ferucarbotran, Gadobutrol, and MnCl_2_) in seven ascending concentrations (5%, 10%, 20%, 40%, 60%, 80%, and 100%) were examined performing three MRI‐sequences (T1 Vibe, T2 TSE, and T1 FLASH). Orientation of the catheters to the main magnetic field was systematically varied between three angulations (0°, 45°, and 90°). Artifact diameters were evaluated in frequency encoding direction (FED) and phase encoding direction (PED), adding up to 378 measurements. All measurements were conducted with axial slice orientation relatively to the catheters (Figure 1).

### 2.3 Data analysis and statistics

Image data were evaluated at a separate workstation using ImageJ (U.S. National Institutes of Health, Bethesda, MD, USA). According to the American Society for Testing and Materials (ASTM), the size of the artifact was defined as the region deviating ± 30% from the median signal intensity around the microtube.^12^ Artifact extents of the filled microtube were measured parallel and orthogonal to the PED.

To compare the artifact size between contrast agents (Gadobutrol vs. Ferucarbotran vs. MnCl_2_), concentrations (5%, 10%, 20%, 40%, 60%, 80%, and 100%), encoding directions (PED vs. FED), and microtube angulations to B_0_ (0°, 45°, and 90°), the values were predicted by these variables and their interactions in a mixed regression model. The three sequences, T1‐VIBE, T2‐TSE, and T1‐FLASH, were treated as a random factor, yielding an estimate of random intercept variance. All other, fixed factors varied within observations. Concentration and microtube angulation were entered as a centered continuous variable (with catheter angulation recoded as 0° = –1; 45° = 0; 90° = +1 for easier interpretation). Contrast agent was treated as a polytomous categorical predictor (effect codes with coefficients E_Fer_:+1;0;–1 and E_Gad_: 0;+1;–1 for Ferucarbotran, Gadobutrol, and MnCl_2_, respectively; E_Fer_ compares Ferucarbotran with MnCl_2_ and E_Gad_ compares Gadobutrol with MnCl_2_). Direction of encoding was used as a dummy variable contrasting FED ( = 0) and PED ( = 1). Differences are reported as regression coefficients with standard errors with mean and standard deviation (SD) for illustration. Regression coefficients with *p* < 0.05 were considered statistically significant. *p*‐values were adjusted following the procedure of Benjamini and Hochberg.^13^ Data were analyzed using R, Version 4.0.3,^14^ particularly the lme4 (Version 1.1‐26)^15^ and sjPlot^16^ (Version 2.8.7) packages.

## 3 RESULTS

**FIGURE 2:**
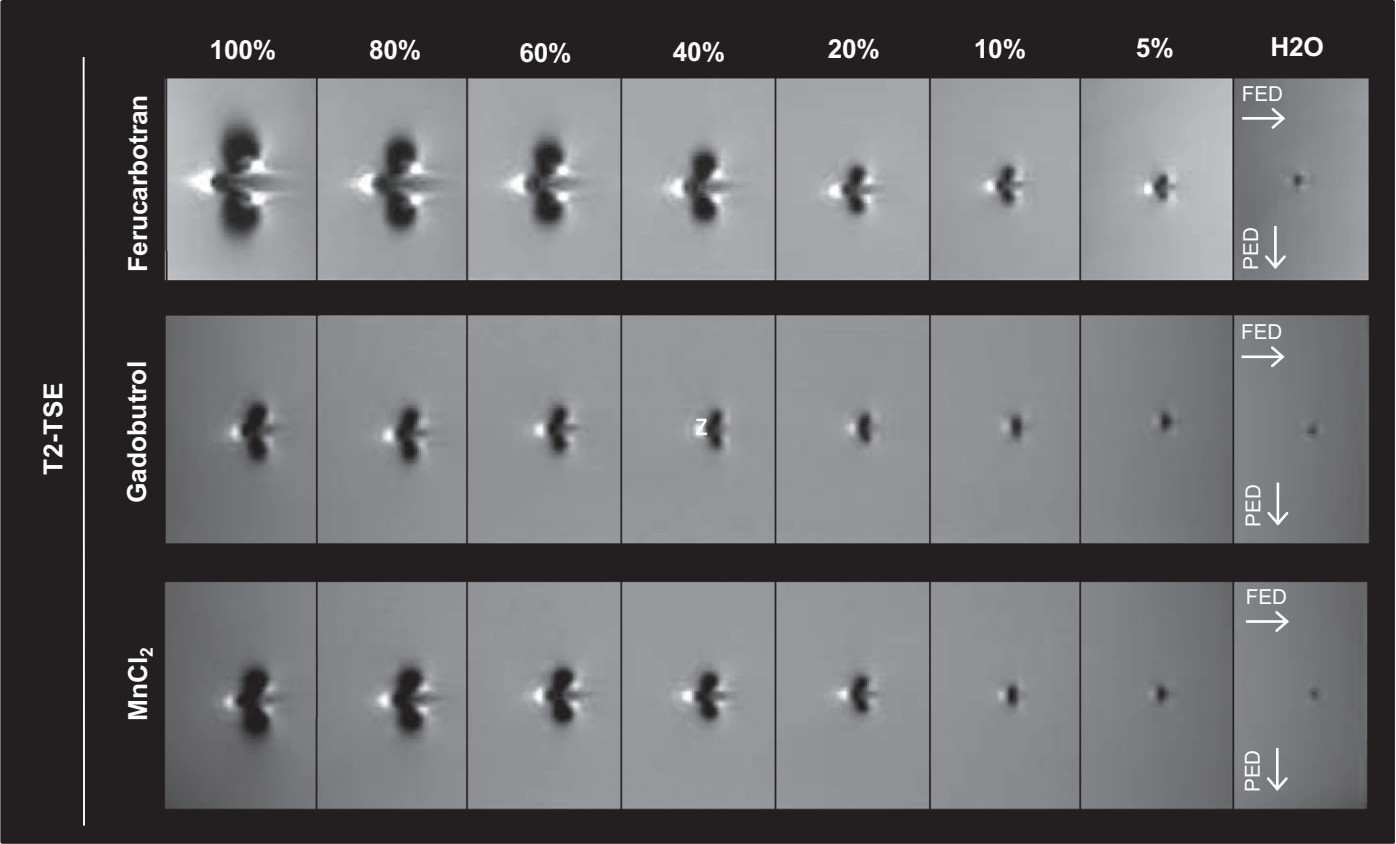
Artifact expansion with respect to different concentrations of Ferucarbotran, Gadobutrol, and MnCl_2_ for the example of the T2‐TSE sequence. Angulation to B0 was 90°, applied contrast agents were Ferucarbotran, Gadobutrol, and MnCl_2_. The expansion of the artifact is dependent on contrast agent, concentration of contrast agent (5–100% of stock solution), and encoding direction. Abbreviations: FED, frequency encoding direction; PED, phase encoding direction.

**FIGURE 3:**
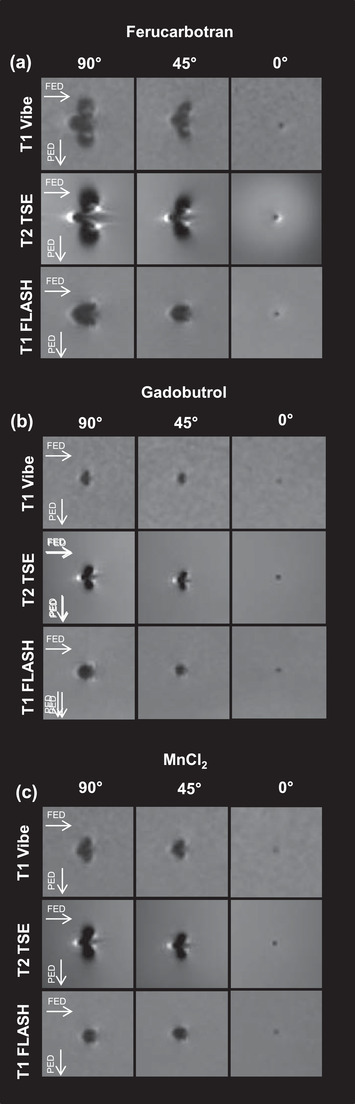
Influence of angulation to B0 on artifact diameter. Angulation of the phantom to B0 was varied between 90°, 45°, and 0°. Applied contrast agents were Ferucarbotran (a), Gadobutrol (b), and MnCl_2_ (c) in 100% stock concentration. Performed MR‐sequences were T1 Vibe, T2 TSE, and T1 FLASH (see text for sequence details). The expansion of the artifact is strongly dependent on angulation to B0 with the largest expansion seen at 90°. Abbreviations: FED, frequency encoding direction; PED, phase encoding direction.

Typical artifact appearance of the phantom is depicted in Figure 2 for the example of the T2‐TSE sequence with respect to different concentrations of Ferucarbotran, Gadobutrol, and MnCl_2_. Typical artifact appearance of the phantom with respect to different angulations is depicted in Figure 3. The typical shape of the artifact was arrowhead or butterfly‐like and symmetric with respect to the FED.

Results are presented in Table 2. Overall, catheter angulation and concentration had effects on the artifact diameters in both PED and FED directions: The greater the angle (b = 2.9, se = 0.09, *p* < 0.001) and the greater the concentration (b = 0.0488, se = 0.0022, *p* < 0.001), the larger the diameters in FED direction. Furthermore, artifact diameters in the FED direction were overall considerably larger with Ferucarbotran compared to MnCl_2_ (b = 4.25, se = 0.21, *p* < 0.001) and smaller with Gadobutrol (b = –3.09, se = 0.21, *p* < 0.001).

**TABLE 2:** Mixed model with all predictors centered and sequence as a random factor

	*Artifact diameter (mm)*		
*Predictors*	*Estimate (CI* ^a^)	*Standard error*	*p*
Intercept	5.289 (4.096; 6.481)	0.608	0.013
Catheter angulation	2.899 (2.720; 3.077)	0.091	<0.001
Concentration	0.049 (0.044; 0.053)	0.002	<0.001
Encoding direction	0.919 (0.628; 1.210)	0.149	<0.001
Ferucarbotran versus MnCl_2_	4.248 (3.836; 4.660)	0.210	<0.001
Gadobutrol versus MnCl_2_	–3.095 (–3.507; –2.683)	0.210	<0.001
Catheter angulation × Concentration	0.037 (0.032; 0.042)	0.003	<0.001
Catheter angulation × Encoding direction	0.860 (0.503; 1.216)	0.182	<0.001
Concentration × Encoding direction	0.018 (0.009; 0.027)	0.004	<0.001
Catheter angulation × Ferucarbotran versus MnCl_2_	2.488 (1.984; 2.993)	0.257	<0.001
Catheter angulation × Gadobutrol versus MnCl_2_	–1.976 (–2.481; –1.472)	0.257	<0.001
Concentration × Ferucarbotran versus MnCl_2_	0.043 (0.030; 0.055)	0.006	<0.001
Concentration × Gadobutrol versus MnCl_2_	–0.033 (–0.045; –0.020)	0.006	<0.001
Encoding direction × Ferucarbotran versus MnCl_2_	0.689 (–0.135; 1.513)	0.420	0.12
Encoding direction × Gadobutrol versus MnCl_2_	–0.879 (–1.703; –0.056)	0.420	0.052
Catheter angulation × Concentration × Encoding direction	0.015 (0.004; 0.026)	0.005	0.01
Catheter angulation × Concentration × Ferucarbotran versus MnCl_2_	0.021 (0.006; 0.036)	0.008	0.01
Catheter angulation × Concentration × Gadobutrol versus MnCl_2_	–0.019 (–0.034; –0.004)	0.008	0.023
Catheter angulation × Encoding direction × Ferucarbotran versus MnCl_2_	1.062 (0.053; 2.071)	0.515	0.053
Catheter angulation × Encoding direction × Gadobutrol versus MnCl_2_	–0.971 (–1.980; 0.037)	0.515	0.071
Concentration × Encoding direction × Ferucarbotran versus MnCl_2_	0.024 (–0.000; 0.049)	0.012	0.068
Concentration × Encoding direction × Gadobutrol versus MnCl_2_	–0.017 (–0.041; 0.008)	0.012	0.19
Catheter angulation × Concentration × Encoding direction × Ferucarbotran versus MnCl_2_	0.019 (–0.011; 0.049)	0.015	0.23
Catheter angulation × Concentration × Encoding direction × Gadobutrol versus MnCl_2_	–0.015 (–0.045; 0.015)	0.015	0.34
Random effects			
Residual variance	2.087		
Random intercept variance _Sequence_	1.094		
ICC	0.344		
*N*_Sequence_	3		
Observations	378		
Marginal *R* ^2^/conditional *R* ^2^	0.806/0.873		

^a^ 95% confidence intervals.

Note: Wald's method is used to compute degrees of freedom. Model is fit by restricted maximum likelihood (REML). *p*‐values were corrected following the procedure of Benjamini–Hochberg.^13^

The effect of the angle was, however, not uniform across concentrations (angle × concentration interaction, b = 0.0425, se = 0.0062, *p* < 0.001): The higher the concentration of the agent, the larger was the effect of the angle. Similarly, the effect of the angle on diameters in the FED direction was more pronounced with Ferucarbotran than with MnCl_2_ (angle × E_Ferobutran_ interaction, b = 2.49, se = 0.26, *p* < 0.001) and less pronounced with Gadobutrol (angle × E_Gadobutrol_ interaction, b = –1.98, se = 0.26, *p* < 0.001). The effects of concentration on diameters in the FED direction were more pronounced with Ferucarbotran (b = 0.04, se = 0.01, *p* < 0.001) and less pronounced with Gadobutrol (b = –0.03, se = 0.01, *p* < 0.001) compared with MnCl_2_.

Finally, the effect of angle on FED directional diameters at higher concentration was greater for Ferucarbotran (angle × concentration × E_Ferobutran_ interaction, b = 0.02, se = 0.01, *p* = 0.006) than with MnCl_2_, and lesser with Gadobutrol (angle × concentration × E_Gadobutrol_ interaction, b = –0.02, se = 0.01, *p* < 0.015).

Overall, artifact diameters were larger in the PED direction than in the FED direction (b = 0.0488, se = 0.0022, *p* < 0.001). In particular, the effects of angle and concentration were more pronounced for measurements in the PED direction (angle × direction interaction, b = 0.86, se = 0.18, *p* < 0.001; concentration × direction, b = 0.0180 (se = 0.0044), *p* < 0.001). The increase of the effect of the angle with increasing concentration was also stronger in the PED direction than the FED direction (angle × concentration × direction interaction, b = 0.02, se = 0.01, *p* < 0.001).The degree to which the effect of angle was more pronounced with Ferucarbotran than with MnCl_2_ was amplified in measurements in the PED direction compared to measurements in the FED direction (b = 1.06, se = 0.51, *p* = 0.039), and descriptively, the degree to which the effect of angle was less pronounced with Gadobutrol than with MnCl_2_ was amplified as well (–0.97, se = 0.51, *p* = 0.059).

**FIGURE 4:**
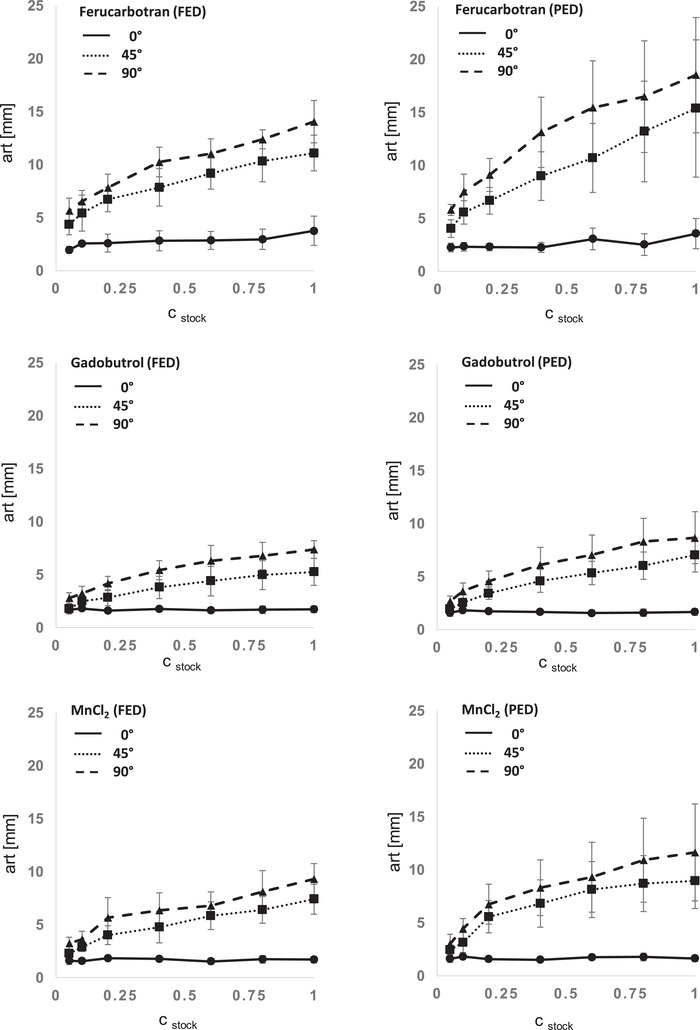
Artifact dimension (art) in relation to contrast medium, concentration of stock solution (c_stock_), and angulation to B0. Graphs display mean artifact dimensions of T1 VIBE, T2 TSE, and T1 FLASH sequences with standard deviation. Abbreviations: FED, frequency encoding direction; PED, phase encoding direction.

Exemplary artifact diameters at the angulation to B0 with the largest expansion (90° angle to B0) and at highest concentration of the stock solution (100%) were 18.5 ± 5.4 mm (PED) and 14.1 ± 2.0 mm (FED) in Ferucarbotran, 8.7 ± 2.5 mm (PED) and 7.4 ± 0.8 mm (FED) in Gadubutrol, and 11.6 ± 4.6 mm (PED) and 9.3 ± 1.5 mm (FED) in MnCl_2_. Artifact dimensions are plotted in Figure 4.

**FIGURE 5:**
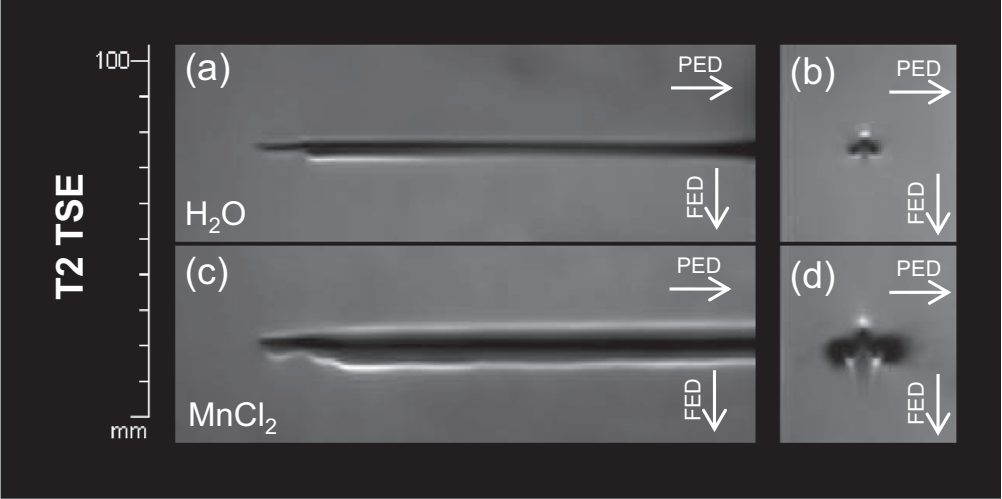
Artifact diameter of MRI compatible microwave therapeutic antennas can be enhanced by filling the cooling circulation system with fluid contrast agent instead of water. Angulation to B0 was 90°, (a) and (c) show coronal, (b) and (d) show axial slice orientation for the example of the T2 TSE sequence. The cooling circulation system was either filled with water (a, b) or 5 mmol/ml MnCl_2_ (c, d).

Figure 5 provides an example for enlargement of artifacts in a routinely used interventional device. MRI compatible microwave therapeutic antennas that were placed in a water‐filled MRI phantom and oriented 90° to B0 caused a homogenous artifact along the shaft and a smaller artifact around the tip. After replacing the water within the cooling circulation system with 5 mmol/ml MnCl_2_, the artifact was enhanced both in T2 TSE (artifact diameter in coronal slice: 7 vs. 13 mm for H2O and MnCl_2_, respectively) and T1 VIBE sequences (artifact diameter in coronal slice: 6 vs. 13 mm for H2O and MnCl_2_, respectively, data not shown).

## 4 DISCUSSION

In this study, we evaluated fluid‐based artifact augmentation of microtubes in MRI that may be transferred to percutaneous interventional devices to temporarily enhance visibility during procedures.

MR‐guided interventions provide the advantage of freely selectable multiplanar needle paths during fluoroscopy that can improve navigation and accelerate device placement in percutaneous interventions.^17^ However, this type of imaging requires a certain visibility of the device.^18^ Since most devices for MRI interventions are made of titanium alloy, they show only limited visibility in MRI. Device visibility under MR‐guidance depends on signal voids and geometrical image distortion induced by magnetic susceptibility.^19^ The expansion of the artifact caused by the device is crucial for the success of the procedure. A small artifact diameter that is only barely visible increases the risk of misplacement as its visibility is impaired especially under the use of MR‐fluoroscopic sequences, whereas large artifacts may obscure anatomical structures and inhibit safe navigation. Performing of control sequences might be helpful, but causes repeated interruptions and lengthens overall procedure time.

It is well known that metallic devices cause local magnetic field inhomogeneities in MRI. Ladd et al. investigated susceptibility artifacts both theoretically and experimentally with respect to different needle materials, needle orientation, and strength of the magnetic field.^20^ Our study mainly focuses on generating transient artifacts on purpose through injection of contrast agents into microtubes. We evaluated artifact augmentation induced by three fluid contrast agents that are commercially available: Gadobutrol (Gadovist®) is an extracellular nonionic macrocyclic gadolinium‐based contrast agent. Due to its diagnostic efficacy and low‐risk profile, it is widely used in clinical routine for contrast‐enhanced MRI and MR angiography.^21^ Ferucarbotran (Resovist®) contains superparamagnetic iron oxide microparticles and was primarily developed for the detection of focal hepatic liver lesions. It contains 0.5 mol Fe/L and induces signal decrease on T2‐weighted images.^22^ However, due to lack of clinical users, it is currently available in only limited countries.^23^ The metal manganese (Mn2+) is paramagnetic and causes strong reduction of both T1 and T2 relaxation times.^24^ Furthermore, it is a trace element for many cellular processes and can be used for functional MRI of the brain. However, it is neurotoxic in excess.^25^ Manganese dichloride is available as an oral contrast agent (LumenHance®) for MRI of the abdomen, but in our study, manganese was purchased as powder and dissolved in water until the required concentration was reached.^26^ When applied in a closed microtube, the utilized contrast agents do not directly interact with the surrounding tissue. Therefore, the relevant property causing artifacts is the magnetic susceptibility of the solutions. The diameter of the artifacts increases with the magnetic susceptibility of the agent and roughly correlates with calculated or estimated values for the applied substances with respect to the dilutions (χ(MnCl_2_) = 14.600 10–6 cgs units, χ(gadopentetic acid, GdDTPA) = 27.200 10–6 cgs units, χ(Fe2O3) = 125.000 10–6 cgs units).^27^, ^28^ In our experiments, all applied fluids were able to increase the artifact diameter in a concentration‐dependent manner. Angulation to B_0_, PED, and FED also had influence on character and expansion of the artifact. In sum, the individual factors as well as their interactions affect artifact diameters in the PED direction in a more pronounced fashion than in the FED direction, where the effects are, however, also present. Similarly, artifacts and interactive effects on them were often larger with Ferucarbotran than with MnCl_2_ and smaller with Gadobutrol than with MnCl_2_.

In the experimental setup, we were able to freely vary the levels of the factors. However, during an intervention, several of these factors are fixed beyond an examiner's control, such as the access route, and therefore, the angulation of the device to B_0_. In most procedures, the interventional device is guided orthogonally to B_0_, therefore, causing the most distinct artifact. At an angulation of 0° to B_0_, we observed no artifact augmentation in our study, so that our proposed fluid‐based augmentation of device visibility is not advantageous for device angulations parallel to the main magnetic field. While the artifact is symmetric with respect to the FED, the largest artifact diameter is obtained in PED direction. In order to maximize artifact diameter also in measurements with parallel slice orientation, PED should be set perpendicular to the device. Theoretically FED and PED can be adjusted as required, but the selection is usually based on the reduction of folding and projection artifacts and not on augmentation of the artifact diameter.^29^ The advantage of a fluid‐based artifact augmentation lies in both the transient and reversible nature and the freely selectable concentration that can be adapted to the requirements of the procedure. According to our clinical experience, an artifact diameter of 8–10 mm is optimal during intervention allowing both safe navigation and visibility without extensive obstruction of anatomical structures. With Ferucarbotran, a mean artifact diameter of 10 mm could be reached at a 90° angle to B_0_ with 20–40% stock concentration (0.1–0.2 mmol/ml). With MnCl_2_, a higher concentration of 80–100% of the stock solution (4–5 mmol/ml) was necessary to achieve a comparable artifact under similar conditions. In Gadobutrol, even the stock concentration of 1 mmol/ml did not induce an artifact diameter of 10 mm (mean diameter 7.4–8.7 mm). Still, Gadobutrol is among the most frequently used contrast media for MRI in clinical routine. Although its artifact diameter is small in comparison, it may, therefore, provide a convenient alternative to enhance visibility of interventional devices.

Most devices being in use for thermal ablations are provided with a cooling circulation system ensuring precise delivery of energy. Usually, an infusion with sterile water is connected to the applicator. Several ways are conceivable for exploiting this feature to enhance the artifact diameter. (1) The coolant could entirely be replaced by contrast agent. However, for economic reasons, this would only be feasible with low‐cost MnCl_2_ due to high‐volume consumption, if using an open system. (2) Using a closed system, replacing the coolant with diluted Gadobutrol might also be possible. (3) The contrast agent is only transiently injected into the applicator for navigation purposes and removed before initiating thermal ablation. In fact, the latter might be the most suitable way for clinical procedures. In MRI‐guided microwave ablation, it is even necessary to unplug the cooling solution and the generator each time the device is moved, since fluoroscopy for navigation purposes is not applicable with the wire attached.

To provide an example of a potential application, we performed measurements using MRI compatible microwave therapeutic antennas after the water within the circulation cooling system was replaced with MnCl_2_. Both in T2 TSE and T1 Vibe sequences, the artifact diameter was enlarged and the visibility was improved. The artifact shape remained similar. When the device is mounted in a water‐filled phantom (as presented in Figure 5), even the artifact of an unmanipulated microwave therapeutic antenna is conspicuous. However, when in‐vivo, the device is surrounded by a less homogenous substance, such as cirrhotic liver parenchyma, it quickly becomes less definable and even a slightly enlarged artifact provides a benefit for the interventionalist. For example, in Figure 5, we additionally conducted measurements with coronal slices, since in clinical interventions, the applicator is usually depicted lengthways and moved approximately 90° to B0.

Our study has several limitations. Experiments were performed with FEP catheters with standardized inside diameters under optimal conditions in an MRI compatible phantom. True in‐vivo signal might deviate from our results depending on the material of the interventional device as well as the surrounding tissue. Our study measurements were achieved with catheters with an inner diameter of 760 μm. Interventional devices, such as microwave antennas and radiofrequency electrodes with a deviating fluid‐shaft diameter, may induce larger or smaller artifacts. Consequently, further studies are required to evaluate the influence of varying fluid‐shaft diameters in specific devices as well as the feasibility under clinical conditions.

Furthermore, it is known that artifact dimensions and shape are also dependent on modification of MR sequence parameters. This study focuses on artifact modification using different contrast media, concentrations, angulations, and encoding directions. However, we did not design this study for statistical evaluation of adjustment of sequence parameters and type, since in clinical interventions, the selection of performed MR sequences during a procedure is mostly limited to the visibility of anatomical structures and pathologies. Further studies are necessary to investigate the improvement of artifact visibility with emphasis on MR sequence modification.

Nevertheless, we demonstrate the application of fluid‐based artifact augmentation in order to enhance visibility of microtubes in MRI. Several variables that determine artifact diameters need to be considered, but concentration of the contrast medium is of utmost relevance as it is the easiest to adjust at will.

## 5 CONCLUSIONS

In this study, we demonstrate the augmentation of MRI artifacts in microtubes through injection of fluid‐based contrast agents. Diameters of the artifact were dependent on type and concentration of the contrast medium, angulation to B_0_, and encoding direction. During MRI‐guided interventions, fluid‐based contrast agents might be applied to interventional devices and thus temporarily adjust the artifact ensuring both visibility and safe navigation.

## CONFLICT OF INTEREST

The authors declare no conflict of interest.

## AUTHOR CONTRIBUTIONS

All listed authors contributed directly and substantially to the intellectual content of the paper.

Kübler, Jens: Drafting the work, data acquisition, interpretation of data, statistical analysis, final approval, and integrity of the work.

Martirosian, Petros: Design of the work, data acquisition, interpretation of data, critical revision, final approval, and integrity of the work.

Jacoby, Johann: Interpretation of data, statistical analysis, critical revision, integrity of the work, and final approval.

Gohla, Georg, Winkelmann, Moritz, and Nikolaou, Konstantin: Interpretation of data, critical revision, final approval, and integrity of the work.

Hoffmann, Rüdiger: Drafting the work, critical revision, statistical analysis, accuracy and integrity, and final approval.

## Data Availability

Data available on request from the authors.
